# Supplementary material for: mRNA and protein expression of fetal insulin receptor in breast cancer cell lines and tissues
Source: Breast Cancer Res Treat. 2026 Jul 8;218(1):2. doi: 10.1007/s10549-026-08018-z (PMC13346282; doi:10.1007/s10549-026-08018-z)
Supplement: Supplementary file 1 — Supplementary Material 1 [file 10549_2026_8018_MOESM1_ESM.docx]

Supplemental Figure 1


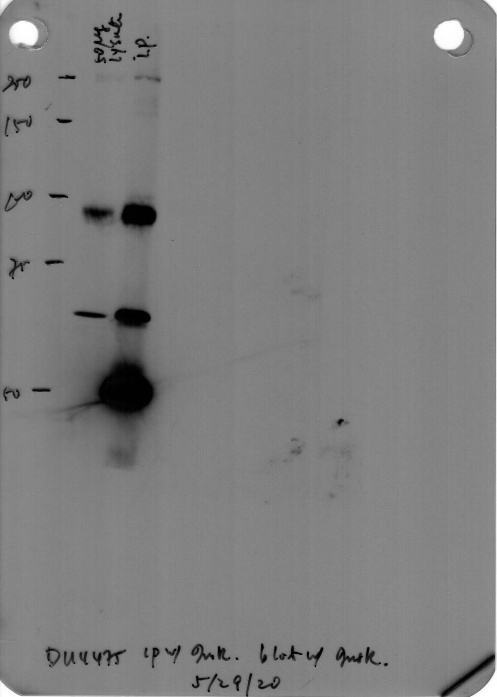


Legend

The complete blot is shown for the edited figure shown in 2A. The left lane represents 50µg of DU4475 cell lysate. The right lane represents 500 μg of lysate protein used for immunoprecipitation with IR antibody.

Supplement Figure 2


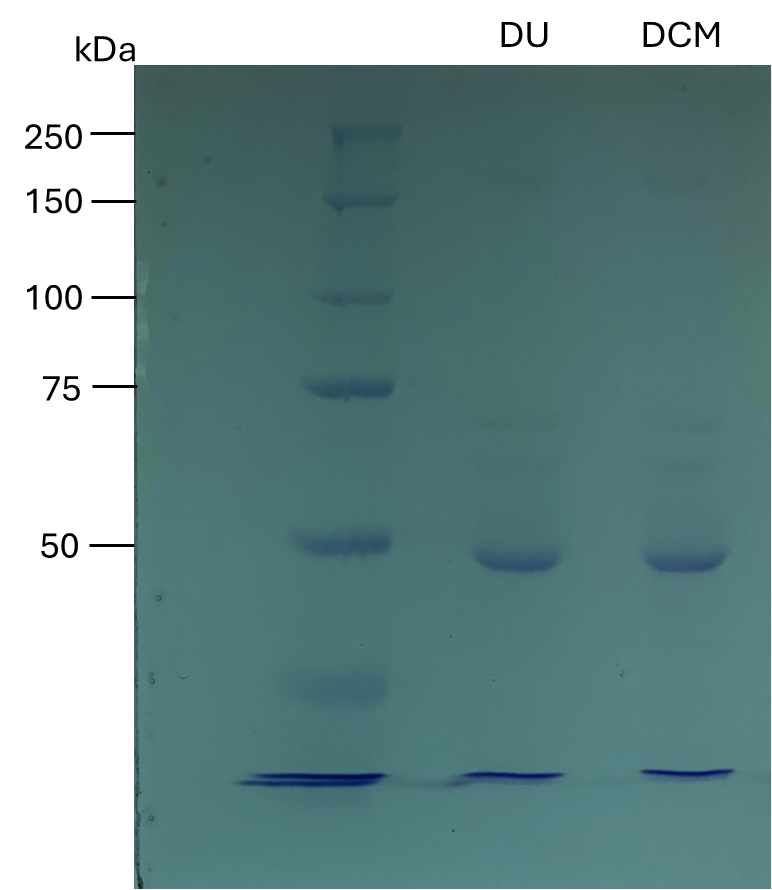


Legend

The complete Coomassie blue gel image is shown for the edited figure shown in 2B. The left lane (DU) represents DU4475 immunoprecipitated membrane protein after PAGE. The right lane (DCM) represents DU4475 and MCF7L (1:1 ratio mixed) immunoprecipitated membrane protein after PAGE. 500 μg of membrane proteins was used for immunoprecipitation with IR antibody.
